# Holo-U^2^Net for High-Fidelity 3D Hologram Generation

**DOI:** 10.3390/s24175505

**Published:** 2024-08-25

**Authors:** Tian Yang, Zixiang Lu

**Affiliations:** 1 School of Computer Science and Technology, Xidian University, South Taibai Road No. 2, Xi’an 710071, China; 23031212364@stu.xidian.edu.cn; 2 Xi’an Key Laboratory of Big Data and Intelligent Vision, Xidian University, South Taibai Road No. 2, Xi’an 710071, China; 3 Guangzhou Institute of Technology, Xidian University, Zhimin Road No. 83, Guangzhou 510555, China

**Keywords:** computer-generated holography, 3D hologram, deep learning, angular spectrum propagation

## Abstract

Traditional methods of hologram generation, such as point-, polygon-, and layer-based physical simulation approaches, suffer from substantial computational overhead and generate low-fidelity holograms. Deep learning-based computer-generated holography demonstrates effective performance in terms of speed and hologram fidelity. There is potential to enhance the network’s capacity for fitting and modeling in the context of computer-generated holography utilizing deep learning methods. Specifically, the ability of the proposed network to simulate Fresnel diffraction based on the provided hologram dataset requires further improvement to meet expectations for high-fidelity holograms. We propose a neural architecture called Holo-U^2^Net to address the challenge of generating a high-fidelity hologram within an acceptable time frame. Holo-U^2^Net shows notable performance in hologram evaluation metrics, including an average structural similarity of 0.9988, an average peak signal-to-noise ratio of 46.75 dB, an enhanced correlation coefficient of 0.9996, and a learned perceptual image patch similarity of 0.0008 on the MIT-CGH-4K large-scale hologram dataset.

## 1. Introduction

Computer-generated holography (CGH), which numerically simulates the interference and diffraction processes of light field propagation to form holographic patterns on a virtual hologram plane, has been applied in various fields, including virtual reality, augmented reality in three-dimensional (3D) displays, and digital holographic microscopy [1]. Various hardware architectures, such as central processing unit-based [2], graphics processing unit-based [3], and field-programmable gate array-based [4] architectures, have been applied to address the trade-off between high-quality holograms and acceptable calculation time. However, generating high-fidelity and real-time holograms remains a significant challenge using traditional methods.

Common methods for generating 3D holograms include the point-based method (PBM) [5], the polygon-based method [6], and the layer-based method [7]. In the PBM, a 3D object is considered to comprise points; points are considered luminous point sources that emit spherical waves that irradiate the hologram plane. The target hologram is obtained by superposing the fringe patterns of all object points on the hologram plane. However, for complicated 3D objects or high-resolution holograms, required the calculation time and memory increase significantly. In the polygon-based method, a 3D object is depicted using polygons that can be considered polygonal apertures. The target hologram is formed by adding the diffraction patterns of all polygonal apertures. In the layer-based method, several layers parallel to the hologram plane represent the 3D object, and the sub-holograms calculated by Fresnel diffraction from each layer are superposed to form the target hologram. However, generating holograms for complicated 3D objects with consistent depths to achieve accurate focal points on the hologram is challenging using these methods, particularly for near-display applications.

Spatial light modulators (SLMs) [8] are essential in holographic display devices for the reconstruction a light field from holograms. An SLM modulates the light field passing through it based on the supplied hologram, which interacts with the reference beam to create an interference pattern. This pattern reconstructs the original object wavefront, enabling the formation of a 3D hologram. The type of SLM used, such as a phase-only SLM, amplitude-only SLM, or complex modulation SLM, significantly influences the characteristics of the generated holograms. Mainstream SLMs cannot manipulate both the amplitude and phase simultaneously. Phase-only holograms (POHs) are commonly employed owing to their higher diffraction efficiency compared to that of amplitude-only modulation. Thus, using a POH as the target hologram is common in hologram tasks. Converting a complex hologram to a POH is effective in iterative methods, such as the Gerchberg–Saxton (GS) method [9], a classic iterative method that propagates complex waves between the object plane and the SLM plane to obtain the intensity distribution and modulating wavefronts with amplitude constraints. However, its lack of phase constraints results in time-consuming computations and speckle noise. Zuo et al. [10] optimized the GS method by introducing a dummy area using an initial quadratic phase and modifying the amplitude constraint strategy. This optimization suppresses speckle noise and improves the quality of the reconstructed image. Peng et al. [11] used the stochastic gradient descent method (SGD) and a camera-in-the-loop optimization strategy to achieve high-quality 2D real-time holographic images in 1080p resolution. Zhang et al. [12] proposed a new non-convex optimization algorithm that computes holograms by minimizing a custom cost function with particular applications or additional information. Chakravarthula et al. [13] revisited complex Wirtinger derivatives to solve the phase retrieval problem and proposed Wirtinger holography-supported lean-based loss functions and SGD methods.

With the rapid advancement of deep learning technology and the increasing capabilities of graphics processing units in parallel computing, deep learning-based methods have shown strong potential for the generation of high-fidelity holograms in real time. Shi et al. [14] utilized an occlusion-aware point-based method to synthesize a large-scale hologram dataset (MIT-CGH-4K) with uniform depth and proposed tensor holography (TensorHolo) to generate complex holograms. They obtained POHs by applying the anti-aliasing double-phase method (AA-DPM), which is an improvement over the double-phase method [15]. The miniaturized TensorHolo achieved hologram generation at 60 fps with TensorRT acceleration. Liu et al. [16] proposed a diffraction model-driven network called DMDNet to synthesize 3840 × 2160 resolution POHs in 0.26 s. DMDNet mainly consists of a UNet-based network [17] combining the residual and sub-pixel convolution methods to generate POHs. A Fresnel diffraction model is also incorporated into the network layer to propagate POHs from the hologram plane to the object plane. Chen et al. [18] proposed a Fourier image network that utilizes residual connections and applies convolutional extraction abilities in both the spatial and Fourier domains, resulting in improved external generalization. Dong et al. [19] proposed a Fourier-inspired neural module and demonstrated its validity using HoloNet [11]. Vision transformers have shown excellent performance in computer vision tasks. As part of state-of-the-art networks, Dong et al. [20] proposed a vision transformer-based holography method utilizing a U-shaped transformer (UFormer) proposed by Wang et al. [21] for image restoration to generate POHs. This method shows promise for computer-generated holography because it modifies the attention mechanism to adapt to holography problems. High-frequency detail preservation and the superposition of sub-holograms involving contextual information can be achieved using U-shaped networks. However, these studies mainly focused on using U-shaped networks to generate complex holograms or POHs, with the full potential of U-shaped networks not yet further explored.

In this study, we propose Holo-U^2^Net to generate high-fidelity 3D holograms based on U^2^Net [22], with its deeper architecture and wider receptive field, achieving high performance on the MIT-CGH-4K large-scale hologram dataset and stimulating the focal effect and defocus blur with a 3D hologram with a real-word capture image.

## 2. Holo-U^2^Net

### 2.1. Overview

U^2^Net was designed for image segmentation tasks, particularly in salient object detection, and has shown competitive results. Compared to the previous UNet structure and its variants, Holo-U^2^Net retains the UNet encoder–decoder structure and multiscale feature extraction ability with a deeper data flow, thereby improving the network’s ability to accurately simulate Fresnel diffraction.

### 2.2. Details of Holo-U*^2^*Net

**Figure 1:**
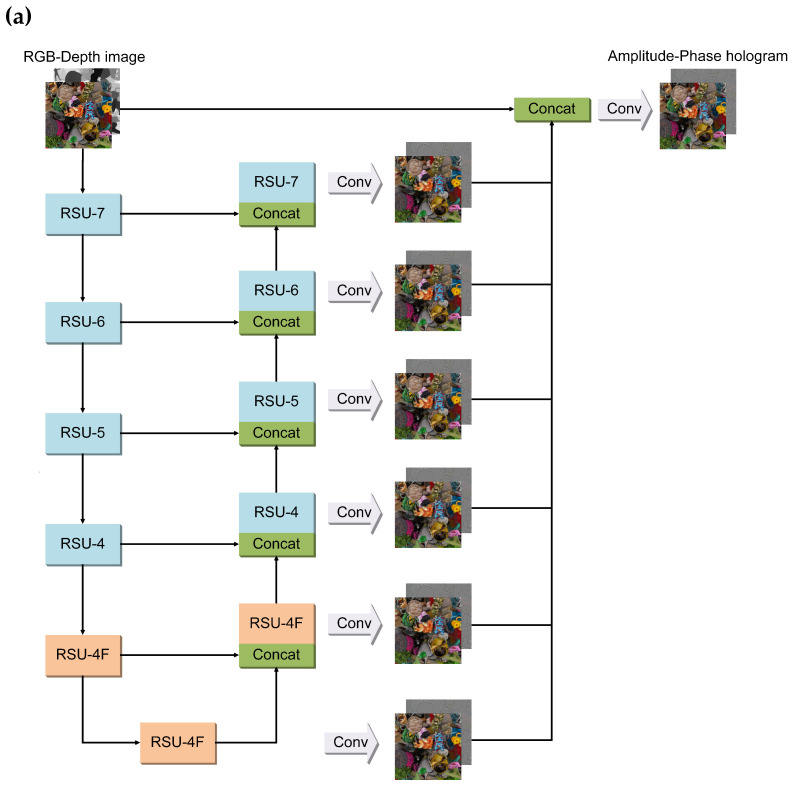

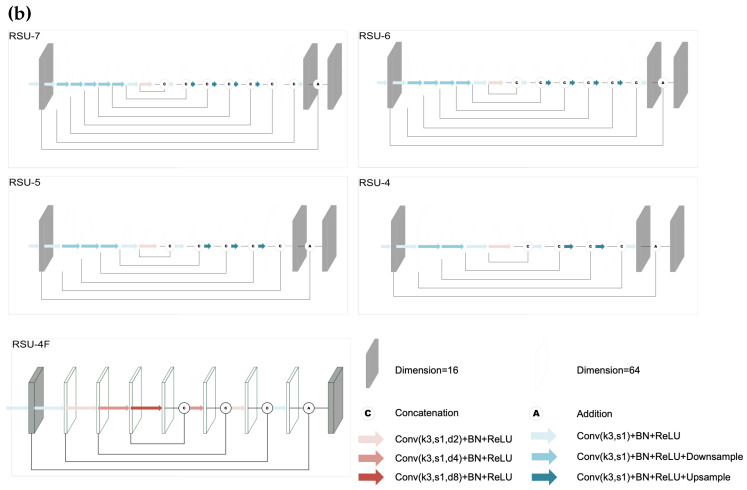
Illustration of the Holo-U^2^Net architecture. Black solid lines in both diagrams indicate the direction of data flow: (**a**) an overview of the Holo-U^2^Net framework, showing the main modules and their arrangement, with RSU-7 to RSU-4 sharing a similar structure and highlighted in the same color to distinguish them from RSU-4F; (**b**) a detailed depiction of Holo-U^2^Net, showing the arrangement of the modules. Feature maps of the same dimension are depicted in the same color.

Holo-U^2^Net is a two-level nested U structure, as illustrated in Figure 1a shows an overview of the main framework and module arrangement, while Figure 1b details the internal structure of each module. The top level consists of a large U structure with 11 residual U blocks (RSUs), while the bottom level refers to a U-shaped structure within each RSU. This nested U structure enables efficient extraction of intrablock multiscale features and aggregation of interblock multilevel features. RSU receives an input feature map denoted as F(x). Its functionality is represented by the U module derived from its U-shaped structure. Utilizing residual connections, the output of the module can be represented as U(F(x)) + F(x).

**Figure 2:**
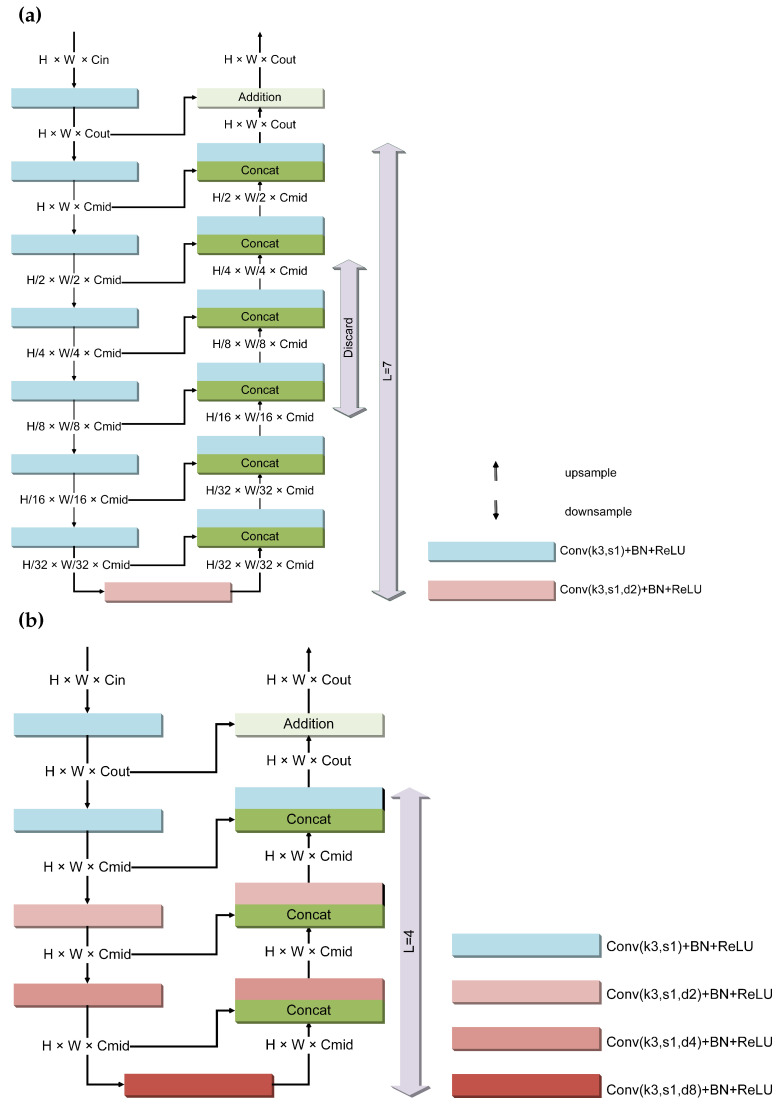
Internal structure of RSU: (**a**) RSU-7; (**b**) RSU-4F. The Conv+BN+ReLU process is represented by rectangular blocks, with blocks of the same color indicating identical convolution parameters. The size of the feature maps obtained from the upper operations is labeled on the solid lines, and the solid lines with arrows indicate the direction of feature map flow in both diagrams.

RSUs exhibit varying heights of 7, 6, 5, 4, and 4F, which can be interpreted as the number of convolutional layers from the feature maps with $C_{\mathrm{out}}$ channels to the bottleneck layer. In Figure 2, we provide a detailed representation of the internal structures of RSU-7 and RSU-4F. H and W denote the current height and width of the feature map, respectively; the parameters “k”, “s”, and “d” in Conv refer to kernel size, stride, and dilation rate, respectively; “BN” stands for batch normalization; the term “Discard” indicates that layers at this level are sequentially discarded as L decreases, corresponding to RSU-6, RSU-5, and RSU-4; and $C_{in}$, $C_{mid}$, and $C_{out}$ represent the current number of channels in the feature map. RSU-4F utilizes dilated convolution and does not involve any upsampling or pooling processes. Thus, intermediate feature maps in RSU-4F have the same resolution as the input feature map. The choice of different heights (L) in RSU-L allows for the extraction of multiscale features from the input feature maps. Larger L values result in deeper RSUs with more pooling operations, larger receptive fields, and richer local and global features [22]. Conversely, smaller L values are used to save memory, reduce computational time, and explore the expressive capacity of shallow layers. Thus, RSU-L provides different ranges of receptive fields and enables the extraction of multiscale features.

In the encoding stage, the number of input channels ($C_{in}$) of RSU-7 is set to 4, while the $C_{in}$ of the other RSUs is set to 64, $C_{mid}$ is set to 16, and $C_{out}$ is set to 64. In the decoding stage, $C_{in}$ is set to 128, $C_{mid}$ is set to 16, and $C_{out}$ is set to 64. The bottom RSU-4F and each RSU in the decoder stage generates feature maps, which are further processed through convolution operations to obtain hologram predictions comprising both amplitude and phase holograms. These predicted holograms are then concatenated with the input RGB-Depth image (RGB-D) and refined through a convolution operation to obtain the final prediction. The average-pooling method is used for downsampling to achieve smoother edges that alleviate artifacts occurring at the object edge. The downsamples and upsamples are discarded in the encoding and decoding stages between the RSUs to retain the repetitive structure and size of the feature maps. The non-linear activation function used in the RSU-block is ReLU. The final predicted hologram is constrained in the output range of [−1, 1] by the Tanh activation function.

### 2.3. Wave Propagation

The Angular Spectrum Method (ASM) [23,24], which decomposes the optical wavefront into plane waves with different spatial frequencies and superimposes them to form a hologram, is suitable for near-field regions for the propagation of wave fields between parallel planes. A specific representation of the ASM is given in Equation (1).

(1) $$U\left(x,y,d\right)=F^{-1}\left[F\left[U(x,y,0)\right]\;\cdot \;\exp\left[i\frac{2\pi }{\lambda }d\sqrt{1-\lambda^{2}\left(u^{2}+v^{2}\right)}\right]\right].$$

The symbol F denotes the Fourier transform operator, and $F^{-1}$ denotes the inverse Fourier transform operator. This equation involves performing a Fourier transform on the complex amplitude of the field (*U*) at position (x,y,0), multiplying it by the transfer function of the ASM that accounts for the frequency (*u*,*v*) and propagation distance (d), then applying the inverse Fourier transform to obtain the field (*d*) at position (x,y,d). Here, λ represents the wavelength of the wave, and i is an imaginary unit. In the experiment, the implementation of the ASM relies on the fast Fourier transform (FFT), inverse fast Fourier transform (IFFT), and frequency in discrete form, as shown in Equation (2).

(2) $$\begin{array}{cc} H_{propagation} & =ASM(H_{origin},d)\\ \; & =IFFT\left[e^{i\frac{2\pi }{d}\sqrt{1-\lambda^{2}\left({\frac{m}{W}}^{2}+{\frac{n}{H}}^{2}\right)}}\;\cdot \;FFT\left[H_{origin}\right]\right], \end{array}$$

where $H_{propagation}$ is the hologram obtained after ASM propagation from the original hologram ($H_{origin}$); $H_{origin}$ is saved in m × n pixels, where m and n correspond to the horizontal and vertical coordinates of the pixel grid, respectively; *M* and *N* denote the width and height of the physical hologram, respectively; *d* is the focusing depth to be propagated; and $d_{\mathrm{in}}$ is selected from the input depth map.

### 2.4. Loss Function

In the training process of Holo-U^2^Net, the loss function was designed based on following two perspectives: the difference between the predicted and target holograms and the difference between the image obtained after propagation and the predicted and target holograms. Therefore, the loss function is defined by Equation (3).

(3) $$L=L_{hologram}+L_{propagation},$$

(4) $$L_{hologram}=\alpha {∥A_{gt}-Ae^{i\left(PhaseDifferenceCorrected)\right)}∥}_{2}+\beta {∥\phi_{gt}-\phi ∥}_{2},$$

(5) $$L_{propagation}=\sigma \;\cdot \;L_{amp}+\epsilon \;\cdot \;L_{tv}.$$

The loss function, denoted as $L_{\mathrm{hologram}}$, calculates the Euclidean norm between the ground-truth hologram and the predicted hologram, as expressed by Equation (4). $A_{gt}$ and $\phi_{gt}$ represent the ground-truth holograms of amplitude and phase, respectively, while *A* and ϕ represent the predicted results of amplitude and phase, respectively, forming the complex predicted hologram. The term PhaseDifferenceCorrected is calculated using the expression $atan2\left(\sin\left(\phi -\phi_{gt}\right),\cos\left(\phi -\phi_{gt}\right)\right)-mean\left(atan2\left(\sin\left(\phi -\phi_{gt}\right),\cos\left(\phi -\phi_{gt}\right)\right)\right)$. This term represents the corrected phase difference between the predicted phase (ϕ) and the ground-truth phase ($\phi_{gt}$). The parameters α and β are set to 1 and 3, respectively.

The loss function ($L_{propagation}$) calculated using Equation (5) includes the total variation loss and L2 loss on the hologram focal stacks calculated by the ASM, simulating free-space wave propagation, as described by Equations (7) and (8). The parameters σ and ϵ are both set to 15. The target hologram and the predicted hologram are propagated to the specified depths, with the selection of these depths being similar to that in TensorHolo. These focal stacks are used to calculate the amplitude images denoted as $AMP_{focal}\left(H,D,d,d_{in}\right)$ in Equation (6). $L_{tv}$ calculates the total variation loss by comparing the focal stacks obtained by propagating the midpoint hologram to different focus depths with the ground-truth hologram propagated to the same depth. The exponential part of the attention mask expressed by Equation (6) assigns higher weights to regions closer to the focusing depth, allowing the network to accurately simulate diffraction in the focused region. The μ parameter, which is utilized to adjust the attention weight of the network between the focus and defocus regions, is set to 0.35 in the experiments. The TV operator represents the calculation of total variation.

(6) $$AMP_{focal}\left(H,D,d,d_{in}\right)=e^{\mu (D-\left(d-d_{in}\right))}\;\cdot \;\left|ASM(H,d_{in})\right|.$$

(7) $$L_{amp}={∥AMP_{focal}\left(H,D,d,d_{in}\right)-AMP_{focal}\left(H_{gt},D,d,d_{in}\right)∥}_{2}.$$

(8) $$L_{tv}=TV\left(AMP_{focal}\left(H,D,d,d_{in}\right)\right)-TV\left(AMP_{focal}\left(H_{gt},D,d,d_{in}\right)\right).$$

## 3. Experiments

### 3.1. Experimental Setup

The settings of the software and hardware environments used in the experiment are listed in Table 1.

### 3.2. Dataset and Metrics

In datasets commonly used for CGH in previous stidues include the DIV2K dataset [25], small-scale datasets, and self-generated datasets without the corresponding holograms. Unsupervised learning methods can address this challenge, albeit with trade-offs in terms of accuracy and quality.

The MIT-CGH-4K dataset is the first large-scale 3D hologram dataset, comprising the following four types of images: rendered, depth, amplitude, and phase images, each with three channels. It includes 4000 images per type, with resolutions of 384 × 384 pixels and 192 × 192 pixels, respectively. These images are generated from random scenes using SLMs with pixel pitches of 8 μm and 16 μm. The dataset is divided into training, validation, and test sets with a ratio of 38:1:1. The complex hologram obtained by combining the amplitude and phase images is a midpoint hologram that applies the wavefront plane concept [26]. This approach optimizes the generation efficiency and memory usage by propagating the original hologram plane to the midpoint plane located at the center of the view frustum to minimize the size of the Fresnel zone plate. The dataset is constructed for a collimated frustum with an optical path length of 6 mm. The position of the midpoint hologram is defined at −3 mm. The playback distance in the simulation is determined based on the depth, which ranges from 0 to 1. Specifically, the playback distance is calculated using the expression −3+6×depth. Compared to other RGB-D datasets [27,28,29], the MIT-CGH-4K dataset exhibits uniform histograms of pixel depth distribution, which benefits network generalization across different depths.

We assessed hologram quality using the following two widely used metrics: the Structural Similarity Index Measure (SSIM) and Peak Signal-to-Noise Ratio (PSNR). SSIM incorporates brightness, contrast, and structural information to quantify the similarity between two images. The closer the SSIM value is to one, the smaller the difference between the images, implying a higher degree of similarity. SSIM is computed using Equation (9).

(9) $$\mathrm{SSIM}\left(x,y\right)=\frac{\left(2\mu_{x}\mu_{y}+C_{1}\right)\left(2\sigma_{xy}+C_{2}\right)}{\left(\mu_{x}^{2}+\mu_{y}^{2}+C_{1}\right)\left(\sigma_{x}^{2}+\sigma_{y}^{2}+C_{2}\right)},$$

where *x* and *y* represent two images; $\mu_{x}$ and $\mu_{y}$ represent the mean values of *x* and *y*, respectively; $\sigma_{x}$ and $\sigma_{y}$ represent the standard deviations of *x* and *y*, respectively; and $\sigma_{xy}$ represents the covariance between *x* and *y*. Here, *x* and *y* refer to two different images, such as amplitude images. Specifically, $\sigma_{x}$ denotes the standard deviation of pixel values in amplitude image *x*, and $\sigma_{y}$ denotes the standard deviation of pixel values in amplitude image *y*. These standard deviations provide insight into the intensity variation within each image, which is important for analyzing the contrast and texture characteristics of the amplitude images. $C_{1}$ and $C_{2}$ are constants used to enhance stability and can be calculated as $C_{1}={\left(K_{1}\;\cdot \;L\right)}^{2}$ and $C_{2}={\left(K_{2}\;\cdot \;L\right)}^{2}$, where *L* represents the data range (in this case, L=1.0). The values for $K_{1}$ and $K_{2}$ are $K_{1}=0.01$ and $K_{2}=0.03$. PSNR, which is based on the mean squared error (MSE) calculated using Equation (10), is commonly used to evaluate differences in quality between the reconstructed and original images, with a higher PSNR value indicating a smaller discrepancy, as expressed by Equation (11).

(10) $$MSE=\frac{1}{mn}\sum_{i=0}^{m-1}\sum_{j=0}^{n-1}{\left[I\left(i,j\right)-K\left(i,j\right)\right]}^{2},$$

(11) $$PSNR=10\;\cdot \;log_{10}{\left(\frac{Max}{MSE}\right)}^{2}.$$

In PSNR calculations, *m* and *n* represent the height and width of the image, respectively; I(i,j) denotes the pixel value of the ground-truth image; and K(i,j) represents the pixel value of the predicted image. The term Max refers to the maximum possible pixel value in an image and is set to 1.0.

In addition to the two commonly used image evaluation metrics mentioned above, we incorporate the use of the Enhanced Correlation Coefficient (ECC) expressed by Equation (12), a method widely employed in image registration and suitable for determining the similarity of two images. Zhang et al. [30] demonstrated that using deep features to assess the perceptual similarity of images bears resemblance to human perception. Therefore, in evaluating performance, we integrate Learned Perceptual Image Patch Similarity (LPIPS), a deep learning-based method for measuring the perceptual similarity of image patches, to assess the similarity of predicted amplitude maps, predicted phase maps, and ground-truth images. The VGG [31] network is utilized to obtain the LPIPS distance.

(12) ECC(H,Hgt)=Re〈Vector(H−H¯)∗,Vector(Hgt−Hgt¯)〉∥Vector(H−H¯)∗∥2∥Vector(Hgt−Hgt¯)∥2,

where *H* and $H_{gt}$ are complex vectors representing processed, predicted, and ground-truth holograms, respectively, after subtracting their respective means; $\langle \mathrm{Vector}{\left(H\right)}^{*},\mathrm{Vector}\left(H_{gt}\right)\rangle$ denotes the inner product of the conjugate transpose of Vector(H) and $\mathrm{Vector}(H_{gt})$; and ${∥\;\cdot \;∥}_{2}$ represents the $L_{2}$ norm; Re denotes the real part of a complex number.

### 3.3. Performance on Holo-U*^2^*Net

To evaluate the performance of these networks in generating 3D holograms, experiments were conducted using the MIT-CGH-4K dataset, which comprises images with a resolution of 192 × 192 pixels. The optical parameters for these experiments included an SLM pitch size of 16 μm; wavelengths of 450 nm, 520 nm, and 638 nm; and holograms with a resolution of 192 × 192 pixels. Holo-U^2^Net was trained for 600 epochs using the Adam optimizer with a learning rate of 1 × 10^−3^ and betas of (0.9, 0.999). We replicated U-shaped networks for hologram generation, including UNet, U^2^Net, and UFormer. Furthermore, we retrained TensorHolo using our experimental setup, following the procedures described in the corresponding paper and open-source code.

Performance evaluation involved computing the amplitude SSIM and PSNR metrics between the ground-truth amplitude image and the predicted amplitude image. Additionally, the focal-stack SSIM and PSNR were calculated for the focal stack obtained by propagating the predicted and ground-truth holograms using Equation (6). This equation enabled the assessment of the reconstructed image quality after ASM propagation. We gathered the results obtained by testing Holo-U^2^Net and the other models on an MIT-CGH-4K test set consisting of 100 samples. The findings are summarized in Table 2. Regarding the SSIM metric based on the predicted and ground-truth amplitude images, the proposed method exhibited the best performance, with an average value of 0.9988, surpassing the second-best model, TensorHolo, by 0.0018. In terms of PSNR, the proposed method achieved an average value of 46.75 dB, surpassing TensorHolo by 3.33 dB. Furthermore, the proposed method exhibited enhanced performance in terms of SSIM and PSNR on the focal stack. The scores on ECC and LPILPS demonstrate the outstanding performance of our network. These results indicate that Holo-U^2^Net can generate high-fidelity 3D holograms.

However, achieving such high-quality results involves trade-offs between quality and computational efficiency. To better evaluate these trade-offs, we set the batch size to 1 and used the MIT-CGH-4K training dataset to train multiple networks, measuring GPU memory usage and Floating Point Operations (FLOPs) during the training process. We further assessed the inference throughput by testing 100 samples from the MIT-CGH-4K test dataset. The results, as detailed in Table 3, offer an assessment of the practical performance of our method. Considering the results reported in Table 2 and Table 3, while our network is not the leader in terms of inference speed or resource usage, it demonstrates a clear advantage in terms of the quality of generated images and meets the real-time computation requirements at the current resolution.

**Figure 3:**
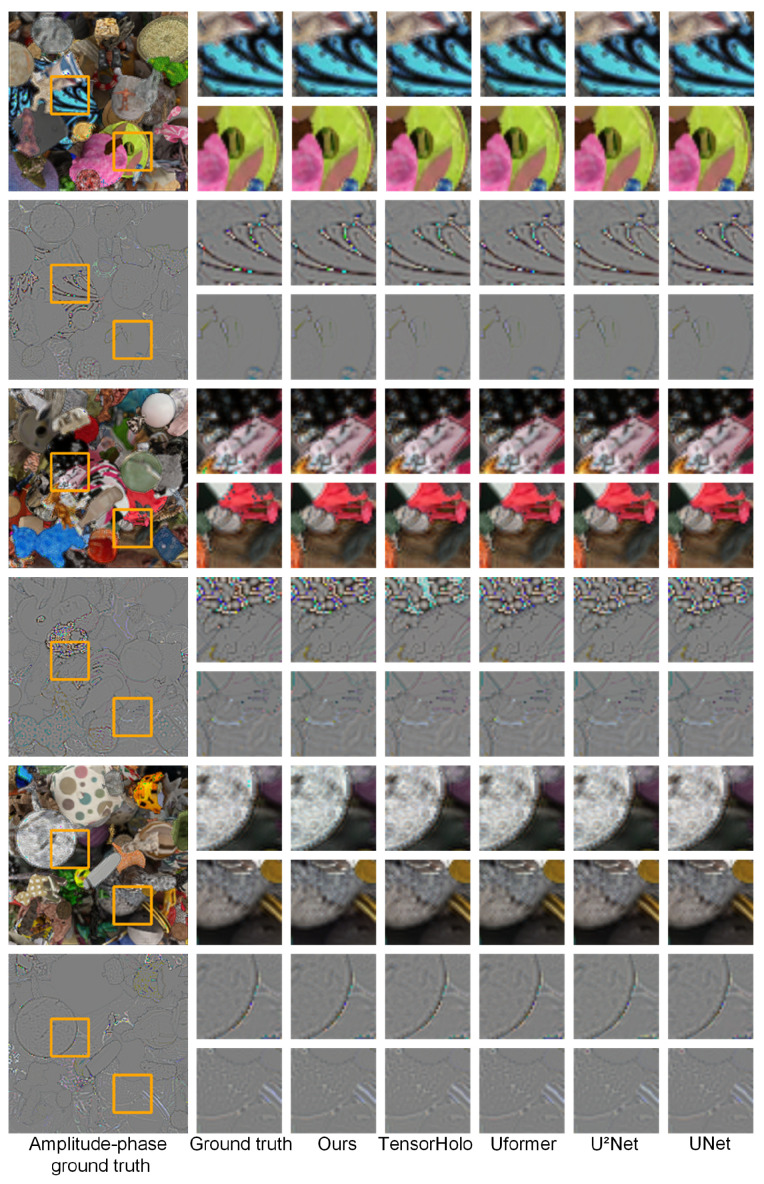
Comparison of amplitude and phase images inferred from three samples using data from MIT-CGH-4K across various networks. The ROIs in the three samples are highlighted with orange rectangular boxes, and the results of the inference from each network are provided on the right side.

Figure 3 displays regions of interest (ROIs) for the amplitude and phase holograms obtained from inference, along with the ground truth, for the first three samples from the MIT-CGH-4K test set. The regions of interest are highlighted with rectangles, and the subsequent columns display the zoomed-in ROIs. Visually, the proposed method produced amplitude and phase holograms that closely resembled the ground truth in terms of object edges, contrast, texture features, and overall fidelity, capturing fine details and preserving structural integrity.

### 3.4. Focus and Defocus Simulation in Holographic Images

We conducted experiments using real-world RGB-D images to simulate the focal effect and defocus blur. Our study primarily focused on exploring networks that demonstrate better image quality performance in generating 3D holograms. Consequently, rather than employing the deep double-phase method [32], which uses a neural network to suppress artifacts and generate phase-only holograms, we adopted the AA-DPM approach to synthesize POHs using Gaussian kernel parameters. To ensure consistency with the parameters employed in TensorHolo, we configured the kernel window size to 3×3 and set the sigma value to 0.7.

**Figure 4:**
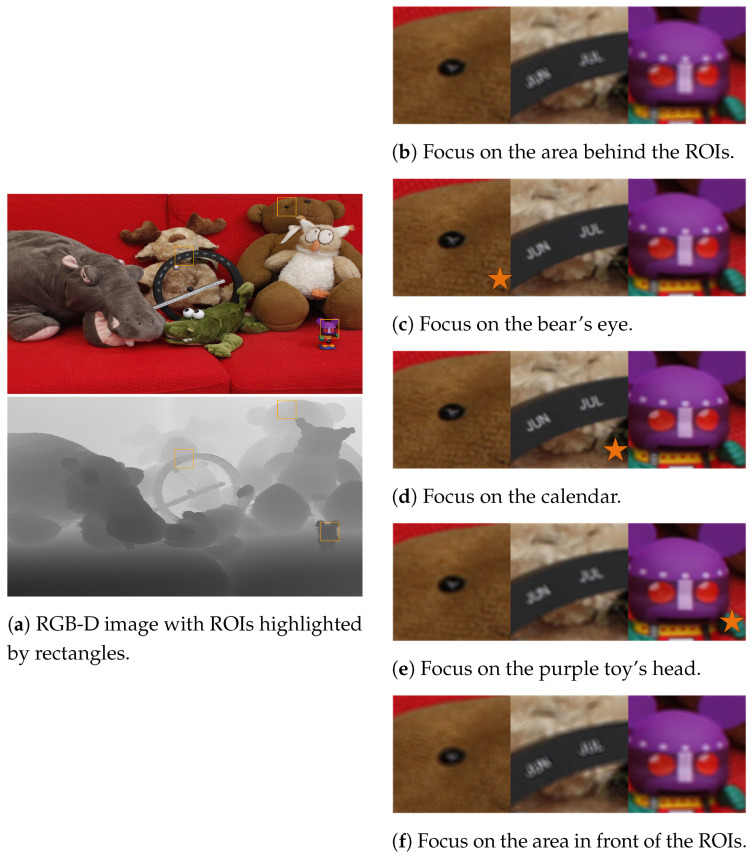
Simulation of focus effects and defocus blur using a real-world RGB-D image, illustrating focus transition from the rear to the front. (**a**) shows the original RGB-D image with ROIs highlighted in orange rectangular boxes. (**b**–**f**) demonstrate the focus effects on various parts of the scene, where orange pentagons indicate the regions that are in focus within the focal stack.

The focal effect and defocus blur based on the reconstructed depth-of-field images are illustrated in Figure 4. The couch image was sourced from a prior study [29]. The region within the orange rectangle is the ROI. In the RGB-D couch image, the focus distance transitions from far to near with a depth shift, resulting in successive focus on the bear’s eye, calendar, and purple toy’s head. The other regions became blurred due to the defocus effect. The ROI is magnified on the right for closer observation, with clearly focused regions indicated by the orange pentagon in the focal stack.

## 4. Conclusions

Deep learning-based computer-generated holography demonstrates notable performance in terms of speed and hologram fidelity. In this study, we enhanced U^2^Net to develop Holo-U^2^Net, an architecture specifically tailored for high-fidelity hologram generation. We carried out experiments on the MIT-CGH-4K hologram dataset. Through extensive validation, Holo-U^2^Net demonstrated an accurate simulation of Fresnel diffraction, resulting in high-quality holograms, as evidenced by its enhanced performance for the SSIM, PSNR, ECC, and LPIPS metrics. Our method achieved better evaluation results than the compared methods. The generated hologram also exhibits better detail representation. The proposed method can achieve complete generalization to generate holograms. In future studies, we will continue to explore methods for accelerating inferences and generalization generation within this framework.

## Figures and Tables

**Table 1:** Software and hardware environments.

Parameter	Details
Operating System	Ubuntu 22.04.04 LTS
Memory	64.0 GiB
CPU	Intel^®^ Core^TM^ i9-14900K
GPU	NVIDIA GeForce RTX 4090 (24 GiB)
Python version	3.8.18
PyTorch version	2.1.2
CUDA version	11.8
Tensorflow version	1.15 (NVIDIA-maintained)

**Table 2:** Performance quality metrics of deep learning networks on the the MIT-CGH-4K test set.

Network	Amplitude SSIM ↑	Amplitude PSNR (dB) ↑	Focal-Stack Amplitude SSIM ↑	Focal Stack Amplitude PSNR (dB) ↑	ECC ↑	LPIPS ↓
UNet	0.9918	38.72	0.9924	38.93	0.9971	0.0065
U^2^Net	0.9922	39.04	0.9925	39.13	0.9974	0.0058
UFormer	0.9956	41.37	0.9963	41.60	0.9985	0.0033
TensorHolo	0.9970	43.42	0.9973	43.53	0.9984	0.0026
Ours	**0.9988**	**46.75**	**0.9988**	**46.94**	**0.9996**	**0.0008**

**Table 3:** Evaluationof efficiency metrics for deep learning networks on the MIT-CGH-4K test set.

Network	FLOPs (GFLOPs)	Inference Throughput (FPS)	GPU Memory Usage (GB)
UNet	30.82	156.373	2.01
U^2^Net	7.34	20.546	5.81
UFormer	6.01	35.340	2.04
TensorHolo	0.06	78.125	1.47
Ours	35.20	54.716	6.51

## Data Availability

The data generated and analyzed in this study are available from the corresponding author on reasonable request.
